# Personal

**Published:** 1910-05

**Authors:** 


### PERSONAL.

Dr. Charles F. Howard, of Buffalo, president of the state board of prisons commissioners, has resigned. Dr. Howard was appointed for a term of four years June 17, 1907. The pay of a commissioner is $10 a day for each day's attendance at meetings, never to exceed $500 in any one year, and actual expenses.

Dr. Howard has been made superintendent of Providence Retreat. His new responsibilities here made it impossible for him to meet the demands upon his time by the constantly increasing work of the state commission.

Dr. J. C. Calhoun of the Buffalo State Hospital staff has gone to New York to take up special work at the Manhattan Eye, Ear and Throat Hospital.

Dr. Sidney D. Wilgus, formerly of Buffalo, has been appointed superintendent of the hospital for the insane at Elgin, Ill.

